# Morphological feminization in hermit crabs (family Paguridae) induced by rhizocephalan barnacles

**DOI:** 10.1186/s40851-025-00252-5

**Published:** 2025-06-05

**Authors:** Asami Kajimoto, Aiko Iwasaki, Tsuyoshi Ohira, Kenji Toyota

**Affiliations:** 1 Department of Biological Sciences, Faculty of Science, Kanagawa University, 3-27-1, Rokkakubashi, Kanagawa-ku, Yokohama-city, Kanagawa 221-8686 Japan; 2 Asamushi Research Center for Marine Biology, Graduate School of Life Sciences, Tohoku University, 9 Sakamoto, Asamushi, Aomori 039-3501 Japan; 3 Department of Biological Science and Technology, Faculty of Advanced Engineering, Tokyo University of Science, 6-3-1 Niijuku, Katsushika-ku, Tokyo, 125-8585 Japan; 4 Department of Bioresource Science, Graduate School of Integrated Sciences for Life, Hiroshima University, 1-4-4, Kagamiyama, Higashihiroshima-shi, Hiroshima, 739-8528 Japan

**Keywords:** Host–parasite interaction, Feminization, Second pleopod, Cheliped length

## Abstract

Rhizocephalans (Thecostraca: Cirripedia) are parasitic crustaceans that infect a wide range of decapod hosts, including hermit crabs, crabs, and shrimps. These parasites exert profound effects on their hosts, inducing parasitic castration, suppressing the development of secondary sexual characteristics, feminizing male crabs, and altering male behavior to resemble that of females. In the present study, we examined the secondary sexual characteristics of two hermit crab species– *Pagurus lanuginosus* from Asari (Hokkaido, Japan) on the Sea of Japan coast and *Pagurus filholi* from Chikura (Chiba, Japan) on the Pacific coast–parasitized by *Peltogasterella gracilis* and *Peltogaster* sp., respectively. Specifically, we assessed the presence of secondary pleopods and the length of the right large cheliped. Our findings demonstrate that male *P*. *lanuginosus* and *P*. *filholi* parasitized by *P*. *gracilis* and *Peltogaster* sp. exhibit morphological changes and characteristics of females, confirming morphological feminization. The magnitude of parasitic effects on morphological feminization varies between the two host species depending on the rhizocephalan genus. Thus, the extent of feminization varies depending on the parasite genus. Notably, different parasite genera induced varying degrees of host modification, even within the same host species. Similarly, the level of feminization caused by a single parasite genus differed between host species. These results highlight the importance of understanding the characteristics of both the hermit crab host and rhizocephalan parasite in developing insights into parasitically induced morphological feminization.

## Introduction

Parasitic infections play a crucial role in marine ecosystems, and can profoundly influence the reproduction and population dynamics of host species [1, 2]. Among marine parasites, rhizocephalans (Thecostraca: Cirripedia) are particularly notable for their infection of various crustaceans, including hermit crabs [3–5], crabs [6–7], and shrimps [8]. These parasites exert a significant impact on their hosts by inducing parasitic castration, thereby eliminating their reproductive capability [9]. Rhizocephalans exhibit highly specialized adaptations to support infection of hosts [10]. Adult females display pronounced sexual dimorphism, hosting dwarf males within their bodies [10–14]. Structurally, the adult female consists of one or more externae (reproductive organs) and an interna, a root-like network that extracts nutrients from the host.

Several rhizocephalan species, especially sacculinids, induce morphological feminization of secondary sexual characteristics in their male crab hosts. This transformation affects abdominal shape, chela size, and copulatory appendages [7, 15–17]. A characteristic modification is the broadening of the male’s normally narrow, semicircular abdomen into a female-like shape, particularly prominent in brachyuran crabs [7, 15–17]. This morphological alteration enables parasitized males to accommodate a greater number of externae within their widened abdomens, facilitating increased offspring hatching [18–20]. Additionally, parasitized male crabs exhibit female-like behaviors, such as larval release activities, involving abdominal waving [21]. Other morphological changes include reduced chela size, modifications to copulatory appendages [7, 16, 22], and alterations in pleopod numbers [16]. Parasitic isopods (Bopyroidea) also induce morphological feminization of secondary sexual characteristics in their hosts [23–26]. However, while some studies suggest that bopyroid infection causes minimal harm to hosts [27], their impact differs from that of rhizocephalans. Unlike rhizocephalans, which chemically castrate their hosts [28], bopyroids impose an energetic burden, leading to reduced reproductive capability [24, 29]. Thus, bopyroid infections are generally considered less harmful, whereas rhizocephalans are known to exert more severe effects on their hosts [30].

Morphological feminization induced by rhizocephalans has been documented not only in crabs but also in anomuran and hermit crabs [31–34]. In hermit crabs, the second pleopod, typically a female-specific trait, is either vestigial or absent in males. However, in *Pagurus samuelis* parasitized by *Peltogaster* sp. and *Pagurus ochotensis* parasitized by *Peltogasterella gracilis*, the second pleopod develops in infected males [31–33]. Additionally, parasitized male *P*. *ochotensis* exhibits reduced right cheliped lengths compared to uninfected males, while this reduction is less pronounced in females [32]. Despite such findings, most studies have focused on single host–parasite pairs, leaving the variation in host effects across different rhizocephalan genera poorly understood.

In this study, we observed the sympatric occurrence of *P*. *gracilis* and *Peltogaster* sp. *P*. *gracilis*, which has previously been reported in Asari and Chikura populations [5, 35, 36], whereas *Peltogaste*r sp. has primarily been documented in adjacent areas, such as Atsuta (Hokkaido, Japan Sea coast) and the Boso Peninsula (Chiba Prefecture, Pacific coast) [3, 37]. The dispersal of rhizocephalans is primarily attributed to the passive distribution of their free-living larvae [38], which may explain how *Peltogaster* sp. is found in the Asari and Chikura populations. Thus, we investigated the effects of some rhizocephalan species, *P*. *gracilis* and *Peltogaster* sp., on hermit crabs from two distinct regions of Japan: *Pagurus lanuginosus* from Asari (Hokkaido, Japan) on the Sea of Japan coast and *Pagurus filholi* from Chikura (Chiba, Japan) on the Pacific coast. We compared the occurrence frequency of the second pleopod and cheliped length between unparasitized (lacking externae) and parasitized male hermit crabs. Then, we assessed the magnitude of parasitic effect on the morphological change in the two host species for each parasite to elucidate the impacts of these parasites on their hosts.

## Materials and methods

### Sample collection

**Fig. 1:**
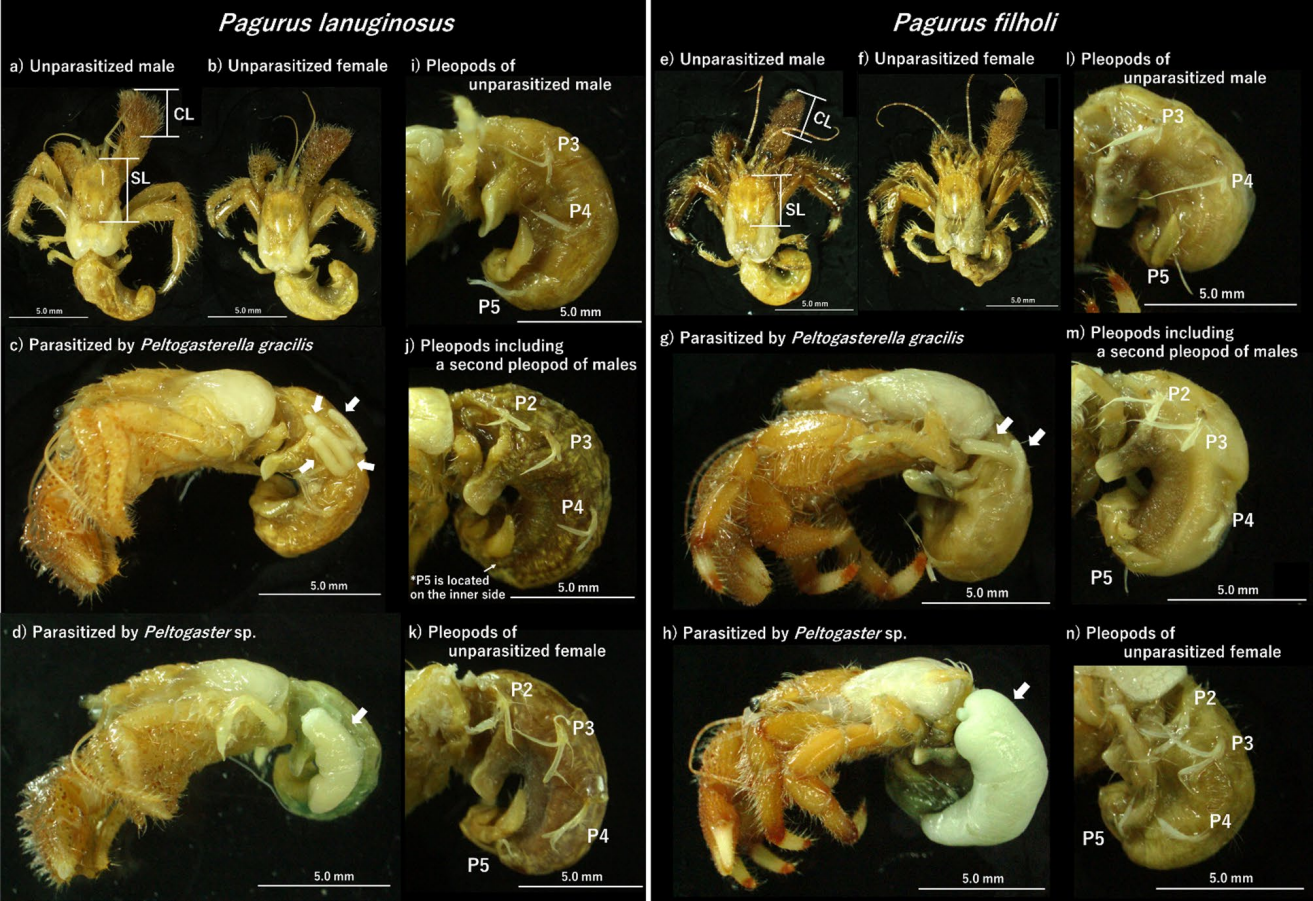
Specimens of host hermit crabs and rhizocephalan parasites used in this study. Unparasitized *Pagurus lanuginosus* male (**a**) and female (**b**). *P*. *lanuginosus* parasitized by *Peltogasterella gracilis* (**c**) or *Peltogaster* sp. (**d**) (white arrows). Unparasitized *Pagurus filholi* male (**e**) and female (**f**). *P*. *filholi* parasitized by *P*. *gracilis* (**g**) or *Peltogaster* sp. (**h**) (white arrows). Pleopods of *P*. *lanuginosus* male without a second pleopod (**i**), with a second pleopod (**j**) and female (**k**). Pleopods of *P*. *filholi* male without a second pleopod (**l**), with a second pleopod (**m**) and female (**n**). CL, cheliped length. SL, shield length. P2, second pleopod. P3, third pleopod. P4, fourth pleopod. P5, fifth pleopod

Unparasitized (lacking externae) *Pagurus lanuginosus* were collected in September 2024 (Fig. 1a, b). Specimens of *P*. *lanuginosus* parasitized by peltogasterellids or peltogastrids were also collected from the shore at Asari, Otaru City, Hokkaido, Japan (43.176°N, 141.068°E) (Fig. 1c and d). Collections were conducted in November 2017, monthly from June to October 2018, and in June, July, September, and November 2019, June, September, and November 2020, September and October 2022, and September 2024. Unparasitized *Pagurus filholi* wer*e* collected in June and October 2024 (Fig. 1e and f). Specimens of *P*. *filholi* parasitized by peltogasterellids or peltogastrids were collected from the coastal area near Chikura, Minamiboso City, Chiba, Japan (34.924◦N, 139.942◦E) in March 2023, as well as in June and October 2024 (Fig. 1g and h). All specimens were preserved in absolute ethanol for subsequent analysis.

### Species identification based on morphological characteristics and cytochrome c oxidase subunit 1 (COI) sequencing

To identify rhizocephalan species, we first observed the morphology of the externae. Peltogasterellids have colonial, elongated externae, with colors ranging from white to yellowish [3, 4]. In contrast, peltogastrids have oval-shaped externae, with colors transitioning from red (immature) to olive and green (mature) [4, 39].

Subsequently, a portion of the externae from peltogasterellids (*n* = 4 from Asari, *n* = 4 from Chikura) and peltogastrids (*n* = 4 from Asari, *n* = 4 from Chikura) was excised for DNA extraction. The samples were taken from randomly selected parasitized hermit crabs preserved in absolute ethanol at room temperature. Genomic DNA was extracted from the tissue samples using a DNeasy Blood and Tissue Kit (Qiagen, Hilden, Germany), following the manufacturer’s protocol. For rhizocephalan species identification, the mitochondrial COI gene regions were amplified by PCR using a primer pair; crust-cox1f (ACTAATCACAAR GAYATTGG) [40] and HCO2198 (TAAACTTCAGGGTGACCAAAAAATCA) [41], under the following conditions: 94 °C for 7 min followed by 35 cycles at 94 °C for 30 s, 45 °C for 30 s, and 72 °C for 2 min, with a final extension at 72 °C for 7 min. TaKaRa Ex Taq or TaKaRa Ex Taq Hot Start Version (Takara, Shiga, Japan) was used for PCR reactions at 50 µL volume. PCR products were treated with a QIAquick PCR Purification Kit (Qiagen, Hilden, Germany). The sequence of each PCR product was verified by DNA sequencing using the Eurofins sequencing service (Eurofins Genomics, Tokyo, Japan). The eight sequences of peltogasterellids and the eight sequences of peltogastrids obtained were deposited in the DNA Data Bank of Japan (DDBJ) under the accession numbers LC865649–LC865664.

### Measurements of morphological traits

The sexes of all hermit crabs were determined by observing the presence of female gonopores under a stereoscopic dissecting microscope. To evaluate the effect of rhizocephalan parasitism on hermit crab morphology, shield length (Fig. 1a and e), and right large cheliped length (Fig. 1a and e) were measured using a digital caliper. Additionally, the presence of a second pleopod was confirmed under a stereoscopic dissecting microscope.

### Statistical analysis

To investigate the effect of rhizocephalan infection on the morphological feminization of male hermit crabs, the frequency of a second pleopod was compared between unparasitized and parasitized males in the Asari and Chikura populations using Fisher’s exact test.

Cheliped length, a secondary sexual characteristic, was used as an indicator of rhizocephalan infection. To assess the effect of parasitism on relative cheliped length in the Asari and Chikura populations, the equality of regression slopes was tested using shield length as a covariate. Statistical analyses were conducted using SAS version 9.4 (SAS Institute Inc., Cary, NC, USA). The following groups were included in the analysis: unparasitized males with or without a second pleopod, unparasitized females, parasitized males with or without a second pleopod, and parasitized females. If no significant interaction between factors was detected, analysis of covariance (ANCOVA) was performed with shield length as a covariate.

For comparisons of the magnitude of parasitic effect on morphological feminization between parasite species for each host species, Hedges’$\:g$ was calculated as a measure of effect size [42]. We calculated the effect sizes as the difference between the mean cheliped/shield length of the parasitized male and unparasitized hermit crabs. We used unparasitized male and female as the baseline for parasitic effects and calculated the effect size for each as follows:

$$\:g=\frac{{X}_{parasitized}-{X}_{unparasitized}}{S}j$$

where $\:S$ is the pooled standard deviation and calculated as

$$\begin{array}{l}\:S=\\\sqrt{\frac{\begin{array}{c}{(N}_{parasitized}-1){S}_{parasitized}^{2}+{(N}_{unparasitized}-1)\:{S}_{unparasitized}^{2}\end{array}}{{N}_{parasitized}+{N}_{unparasitized}}}\end{array}$$

Here, $\:{S}_{parasitized}$and $\:{S}_{unparasitized}$ are the standard deviations of cheliped/shield length in parasitized and unparasitized groups, respectively. $\:j$ is a weighting factor based on the number of individuals ($\:N$) in each case, for the two groups, and is calculated as follows:

$$\:j=1-\frac{3}{{4(N}_{parasitized}+{N}_{unparasitized}-2)-1}$$

A 95% confidence interval (CI) was generated using bootstrapping procedures over 2,000 iterations [43]. Negative values of the effect size in x-axis and y-axis denote smaller cheliped/shield length compared to unparasitized male and female and positive values denote larger cheliped/shield length, vice versa. When the CI does not include zero, it indicates a statistically significant effect size. We conducted the analyses for effect size using R (version 4.2.2; R Core Team, 2022) and the following R packages: ‘ggplot2’ (version 3.4.4) for data visualization, ‘dplyr’ (version 1.1.3) for data manipulation, and ‘BootES’ (version 1.3.0) for bootstrapping procedures.

## Results

### Occurrence of hermit crabs with peltogasterellids or peltogastrids

In the Asari population, 65 males and 88 females were unparasitized (externa-free), while 171 males and 207 females were infected with peltogasterellids, and 11 males and 12 females with peltogastrids. In the Chikura population, 30 males and 41 females were unparasitized, while 42 males and 32 females were parasitized by peltogasterellids, and 47 males and 45 females by peltogastrids.

### Rhizocephalan species identification

Cytochrome c oxidase subunit 1 sequencing identified all eight peltogasterellids studied as *Peltogasterella gracilis*, based on morphology and sequence similarity (98.44–100%) with reference data (accession numbers: MK604154, OR481992). Among the peltogastrids, those from Asari showed 98.95–100% identity with *Peltogaster* sp. (accession numbers: OR481986, OR481989), while those from Chikura included three individuals matching *Peltogaster postica* (99.67–99.84%, MK604147) and one matching *Peltogaster lineata* (99.01%, MK604142). Due to the lack of prior studies on *Peltogaster* sp. parasitizing hermit crabs in Asari and Chikura, its identification was limited to the genus level.

### Presence of the second pleopod in parasitized male hermit crabs

The elongated second pleopod is a female-specific morphological characteristic of *P*. *lanuginosus* and *P*. *filholi* (Fig. 1k and n), typically present only as a vestigial structure in unparasitized *P*. *lanuginosus* males and *P*. *filholi* males (Fig. 1i and l). However, a second pleopod was observed in *P*. *lanuginosus* and *P*. *filholi* males parasitized by *P*. *gracilis* or *Peltogaster* sp. in this study (Figs. 1j and m). Among male *P*. *lanuginosus*, individuals parasitized by *P*. *gracilis* exhibited a significantly higher frequency of a second pleopod compared to unparasitized individuals (Table 1; Fisher’s exact test, *p* < 0.01). Conversely, males parasitized by *Peltogaster* sp. had a significantly lower frequency of a second pleopod compared to those parasitized by *P*. *gracilis* (Table 1; Fisher’s exact test, *p* < 0.01).

**Table 1:** Number of unparasitized or parasitized male and female *Pagurus* individuals, with or without the second pleopod

Sampling site	Hermit crab			No. of individuals		
	Species	Type		without the second pleopod	with the second pleopod	
Asari (Hokkaido)	*Pagurus lanuginosus*	Unparasitized male		61	4	
		Male parasitized by *P. gracilis*		106	65	
		Male parasitized by *Peltogaster* sp.		9	2	
		Unparasitized female		0	88	
Chikura (Chiba)	*Pagurus filholi*	Unparasitized male		40	10	
		Male parasitized by *P. gracilis*		16	26	
		Male parasitized by *Peltogaster* sp.		46	1	
		Unparasitized female		0	41	

Similarly, among male *P*. *filholi*, the frequency of a second pleopod was significantly higher in individuals parasitized by *P*. *gracilis* compared to unparasitized individuals (Table 1; Fisher’s exact test, *p* < 0.01). Moreover, males parasitized by *Peltogaster* sp. exhibited a substantially lower frequency of a second pleopod compared to those parasitized by *P*. *gracilis* (Table 1; Fisher’s exact test, *p* < 0.01).

### Allometric variation in cheliped length between unparasitized and parasitized hermit crabs

**Fig. 2:**
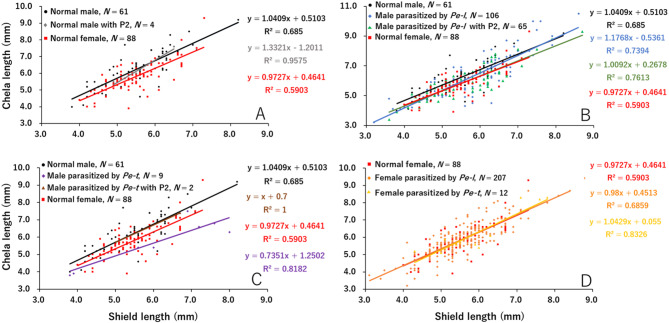
Allometric variation in cheliped length of *Pagurus lanuginosus* among unparasitized males and females (**A**), males parasitized by *P*. *gracilis* (*Pe-l*) (**B**) or *Peltogaster* (*Pe-t*) sp. (**C**), and females parasitized by *Pe-l* or *Pe-t* (**D**). P2, second pleopod

**Fig. 3:**
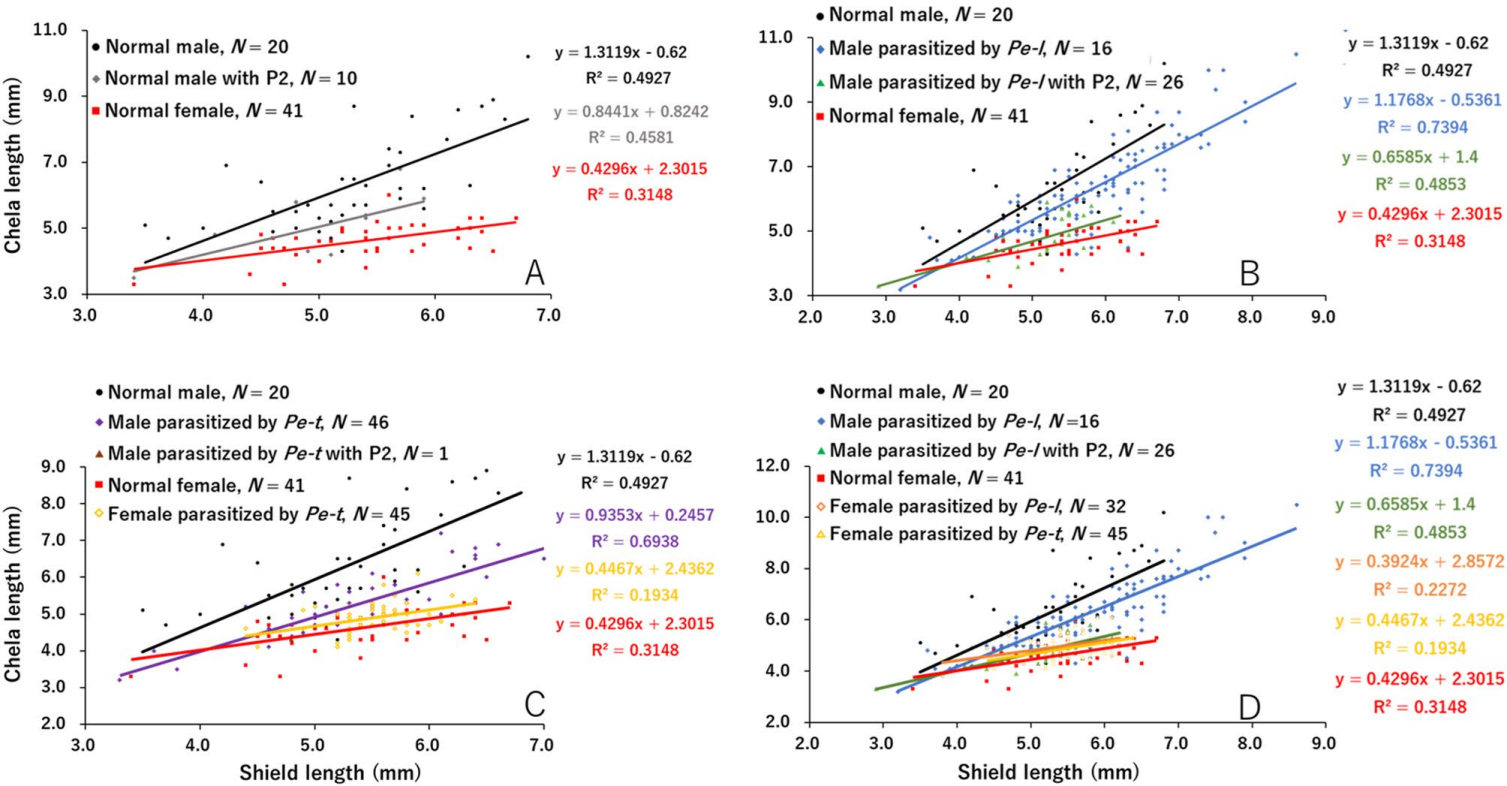
Allometric variation in cheliped length of *Pagurus filholi* among unparasitized males and females (**A**), males parasitized by *Peltogasterella gracilis* (*Pe-l*) (**B**) or *Peltogaster* (*Pe-t*) sp. (**C**), and females parasitized by *Pe-l* or *Pe-t*, as well as males parasitized by *Pe-l* (**D**). P2, second pleopod

In both host species, *P*. *lanuginosus* and *P*. *filholi*, the right cheliped length of individuals parasitized by *P*. *gracilis* or *Peltogaster* sp. was significantly smaller than that of unparasitized ones and more similar to that of females (Figs. 2 and 3). Distinct differences were observed in the relationship between shield length and right cheliped length in unparasitized male and female *P*. *lanuginosus* (Table 2a; Fig. 2A). For the regression lines of unparasitized males with a second pleopod, no significant differences were detected compared to those of unparasitized males and females (Table 2b; Fig. 2A). Similarly, the right cheliped in males parasitized by *P*. *gracilis*, without or with a second pleopod, showed clear reductions in length when compared to those of unparasitized males (Table 2c, d; Fig. 2B). The regression lines of males parasitized by *P*. *gracilis* closely resembled those of unparasitized females particularly, for parasitized males with a second pleopod, whose regression lines overlapped with those of unparasitized females (Fig. 2B). In contrast, males parasitized by *Peltogaster* sp., regardless of the presence of a second pleopod, showed no significant differences in cheliped length compared to unparasitized males, although the small sample sizes limited the statistical power of this finding (Table 2e, f; Fig. 2C). Among *P*. *lanuginosus* females, no significant differences in cheliped length were detected between unparasitized individuals and those parasitized by either *P*. *gracilis* or *Peltogaster* sp., with their regression lines overlapping (Table 2g, h; Fig. 2D).

**Table 2:** ANCOVA of cheliped length in unparasitized male and female *Pagurus lanuginosus* versus those parasitized by *Peltogasterella gracilis* or *Peltogaster* sp.

Comparison of the regression lines	Homogeneity of the slopes of the regression lines		Allometric variation in cheliped length	
	F value	*p*-value	F value	*p*-value
a. Unparasitized male vs.				
Unparasitized female	0.29	0.589	19.180	< 0.0001
b. Unparasitized male with the second pleopod vs.				
Unparasitized male	0.29	0.590	0.05	0.829
Unparasitized female	0.55	0.460	1.70	0.195
c. Male parasitized by *P. gracilis* vs.				
Unparasitized male	1.26	0.263	6.61	< 0.05
Unparasitized female	2.90	0.090	1.97	0.163
d. Male parasitized by *P. gracilis* with the second pleopod vs.				
Unparasitized male	0.08	0.784	16.06	< 0.001
Male parasitized by *P*. *gracilis*	2.53	0.114	2.10	0.150
Unparasitized female	0.11	0.746	0.00	0.946
e. Male parasitized by *Peltogaster* sp. vs.				
Unparasitized male	0.13	0.716	3.39	0.070
Unparasitized female	0.60	0.439	0.01	0.931
f. Male parasitized by *Peltogaster* sp. with the second pleopod vs.				
Unparasitized male	0.01	0.938	0.01	0.911
Male parasitized by *Peltogaster* sp.	0.03	0.862	0.43	0.529
Unparasitized female	0.00	0.953	1.02	0.317
g. Female parasitized by *P*. *gracilis* vs.				
Unparasitized female	0.01	0.941	0.15	0.696
h. Female parasitized by *Peltogaster* sp. vs.				
Unparasitized female	0.17	0.684	0.00	0.989

In *P*. *filholi*, a significant difference was observed in the slopes of regression lines for cheliped length between unparasitized males and females (Table 3a; Fig. 3A). For unparasitized males with a second pleopod, significant differences in cheliped length were found compared to both unparasitized males and females, with their regression line being closer to that of the females (Table 3b; Fig. 3A). Clear differences in cheliped length were also observed between *P*. *filholi* males parasitized by *P*. *gracilis* and unparasitized individuals, with the regression line of parasitized males approaching that of females (Table 3c; Fig. 3B). For males parasitized by *P*. *gracilis* and bearing a second pleopod, the slope of the regression line differed significantly from that of unparasitized males (Table 3d; Fig. 3B). Additionally, significant differences in cheliped length were observed compared to unparasitized females, although no differences were detected when compared to males parasitized by *P*. *gracilis* that did not develop a second pleopod (Table 3d; Fig. 3B). Cheliped length in males parasitized by *Peltogaster* sp. also differed significantly from that of unparasitized males (Table 3e; Fig. 3C). The slope of the regression line for males parasitized by *Peltogaster* sp. differed significantly from that of unparasitized females (Table 3e; Fig. 3C). Although the small sample size hindered precise analysis of *P*. *filholi* males parasitized by *Peltogaster* sp. with a second pleopod, their cheliped length overlapped with that of females parasitized by *Peltogaster* sp. (Fig. 3C). For *P*. *filholi* parasitized by either *P*. *gracilis* or *Peltogaster* sp., the regression lines were parallel to those of unparasitized females, with significant differences in cheliped length observed between these groups (Table 3f, g; Fig. 3D).

**Table 3:** ANCOVA of cheliped length in unparasitized male and female *Pagurus filholi* versus those parasitized by *Peltogasterella gracilis* or *Peltogaster* sp.

Comparison of the regression lines	Homogeneity of the slopes of the regression lines		Allometric variation in cheliped length	
	F value	*p*-value	F value	*p*-value
a. Unparasitized male vs.				
Unparasitized female	13.25	< 0.001	-	-
b. Unparasitized male with a second pleopod vs.				
Unparasitized male	0.99	0.3241	7.13	< 0.05
Unparasitized female	2.74	0.1047	11.46	< 0.0001
c. Male parasitized by *P*. *gracilis* vs.				
Unparasitized male	1.76	0.1908	14.52	< 0.001
Unparasitized female	3.26	0.0768	12.55	< 0.0.001
d. Male parasitized by *P*. *gracilis* with the second pleopod vs.				
Unparasitized male	4.50	< 0.05	-	-
Male parasitized by *P*. *gracilis*	0.38	0.5424	0.84	0.3656
Unparasitized female	1.82	0.1827	38.85	< 0.0001
e. Male parasitized by *Peltogaster* sp. vs.				
Unparasitized male	2.82	0.0971	45.52	< 0.0001
Unparasitized female	13.26	< 0.001	-	-
f. Female parasitized by P. gracilis vs.				
Unparasitized female	0.05	0.828	12.15	< 0.001
g. Female parasitized by *Peltogaster* sp. vs.				
Unparasitized female	0.01	0.924	6.53	< 0.0001

### Comparison of the parasitic effect on morphological feminization between parasite species for *P*. *lanuginosus* and *P*. *filholi*

**Fig. 4:**
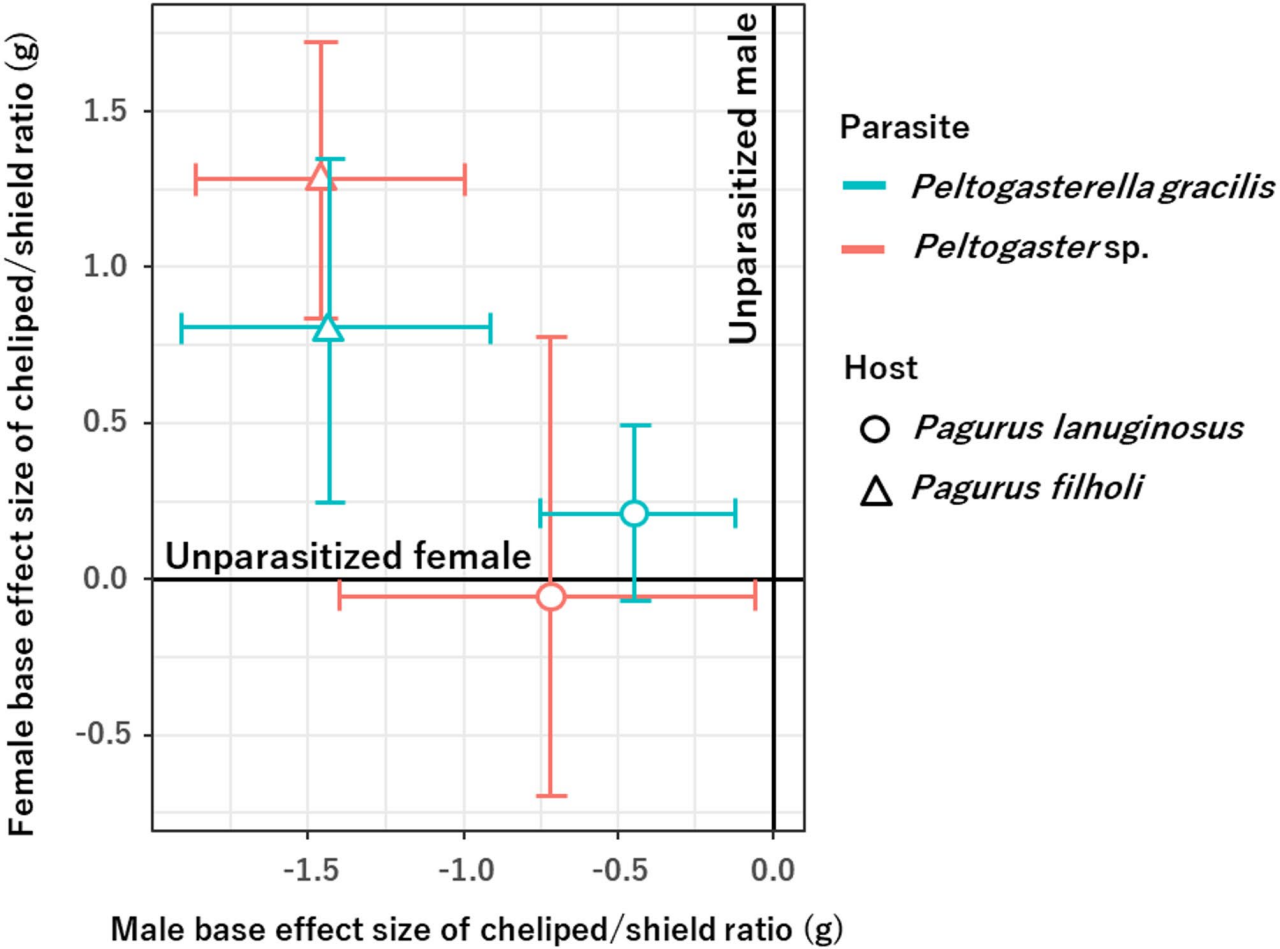
Effect sizes of cheliped/shield length ratios between *Pagurus lanuginosus* and *Pagurus filholi* parasitized by *Peltogasterella gracilis* or *Peltogaster* sp. compared to unparasitized *P*. *lanuginosus* and *P*. *filholi* males (y-axis) and females (x-axis), respectively

Although the cheliped/shield length ratios of males parasitized by *P*. *gracilis* or *Peltogaster* sp. were significantly smaller than those of unparasitized males (x-axis) in both hermit crab species, *P*. *lanuginosus* and *P*. *filholi*, the effect size of morphological feminization (negative value on the x-axis, deviation from 0 on the y-axis) varied among parasite species depending on host species (Fig. 4). The magnitude of the negative parasitic effect on the cheliped/shield length ratio was more pronounced in *P*. *filholi* compared to *P*. *lanuginosus* when compared to unparasitized males (x-axis), while the cheliped/shield length ratio was more similar to that of unparasitized females (y-axis) in *P*. *lanuginosus* than in *P*. *filholi*. Among parasite species, for *P*. *lanuginosus* the negative effect on cheliped/shield length ratios relative to unparasitized males was greater in *Peltogaster* sp. than in *P*. *gracilis for P. lanuginosus*, whereas for *P*. *filholi* the effect was similar between the two parasites. In comparison to unparasitized females, the cheliped/shield length ratios in *Peltogaster* sp. were more similar to those of unparasitized females (y = 0) than in *P*. *gracilis* for *P*. *lanuginosus*, however, a contrasting pattern was observed in *P*. *filholi* (Fig. 4).

## Discussion

### Presence of a second pleopod in males parasitized by *P*. *gracilis* or *Peltogaster* sp.

The presence of the second pleopod, absent in unparasitized males (Fig. 1i and l), was observed in males of both *P*. *lanuginosus* (Fig. 1j) and *P*. *filholi* (Fig. 1m), indicating that parasitized males exhibit morphological changes resembling female-specific characteristics. This observation is consistent with previous reports by Shiino 1931 [31], Oguro 1955 [32], and Nielsen 1970 [33]. The transformation of pleopod composition suggests that parasitized males develop structures resembling the egg-carrying appendages of females, likely to protect and support rhizocephalan externae. The frequency of second pleopod appearance in males parasitized by *P*. *gracilis* was consistently higher than in those parasitized by *Peltogaster* sp. (Table 1), indicating that the extent of parasitic influence varies among the rhizocephalan genus. While previous studies have merely noted the presence of a second pleopod in hermit crabs parasitized by peltogasterellids or peltogastrids, the present study provides a detailed comparison of the frequency of second pleopods observed in *P*. *lanuginosus* and *P*. *filholi* hosts infected by *P*. *gracilis* or *Peltogaster* sp. One distinguishing feature between these parasites is the number and morphology of externae: *P*. *gracilis* typically exhibits multiple, elongate externae (Fig. 1c and g), whereas *Peltogaster* sp. possesses a single, oval-shaped externa (Fig. 1d and h) [3, 4], a pattern also observed in this study.

Grooming behavior by hosts is critical for maintaining rhizocephalan externae, as undergroomed externae can become fouled and necrotic [44]. In *P*. *gracilis*, which produces multiple externae, the presence of a second pleopod in male hosts may facilitate grooming. However, second pleopods were also observed in some unparasitized males (Table 1). In the hermit crab genera *Paguristes* and *Pseudopaguristes*, which have no reported cases of rhizocephalan parasitism, the absence of the second pleopod in males has been observed [45, 46]. The presence of a second pleopod in some males without externae may indicate previous rhizocephalan infection. Such individuals may have lost their externae, or could be in the early stages of infection, where the interna is developing before the formation of the externae. To confirm the actual prevalence of parasitism, dissections of host abdomens and DNA barcoding analysis of rhizocephalan interna are necessary.

### Reduction of cheliped length in males parasitized by *P*. *gracilis* or *Peltogaster* sp.

In *P*. *lanuginosus* and *P*. *filholi* males parasitized by *P*. *gracilis*, cheliped lengths were significantly reduced compared to unparasitized individuals, regardless of the presence of a second pleopod (Tables 2c, d and 3c, d; Figs. 2B and 3B). This indicates a clear morphological feminization effect, consistent with previous findings [32]. In contrast, males parasitized by *Peltogaster* sp. showed no reduction in cheliped length in *P*. *lanuginosus* (Table 2e, f; Fig. 2C), although reductions were observed in *P*. *filholi* (Table 3e, f; Fig. 3C). Furthermore, *P*. *filholi* parasitized by *P*. *gracilis* had a significantly smaller cheliped length compared to *P*. *lanuginosus* parasitized by *P*. *gracilis* (Fig. 4); the parasitic effect size differed between host species. In contrast, no such difference in terms of cheliped size reduction was observed between *P*. *lanuginosus* and *P*. *filholi* parasitized by *Peltogaster* sp. (Fig. 4); the parasitic effect sizes were similar in both host species. These findings highlight the genus-specific impact of rhizocephalans on their hosts. Notably, different parasite genera induced varying degrees of host modification, even within the same host species. Similarly, the level of feminization caused by a single parasite genus differed between host species, and some rhizocephalan species, such as *P*. *gracilis* in this study, exhibited differences in the parasitic effect size on the host. Nielsen 1970 [33] also noted that the extent of morphological changes varies among rhizocephalan species and that the same parasite species may exert different effects depending on the host species. More specifically, Nielsen (1970) [33] reported species-specific parasitic effects of *Peltogasterella sulcata* and *Peltogaster paguri* on various hermit crab hosts, based on morphological changes, such as the presence or modification of the second pleopod. Moreover, subsequent studies to date have offered only limited quantitative analyses of specific host–rhizocephalan parasite interactions [7]. In contrast, the present study defines morphological feminization using quantifiable traits, including the frequency of second pleopods and reduction of chelipeds, and demonstrates that the intensity of these effects varies depending on the specific host–parasite combination. However, in interspecific analysis of the host species, the cheliped length of parasitized *P*. *lanuginosus* was closer to that of females than that of parasitized *P*. *filholi*, regardless of the parasite species (Fig. 4). These quantitative effect-size contrasts (Fig. 4) clearly demonstrate how our results extend earlier qualitative descriptions by Nielsen (1970) [33] and others, underlining the novelty of the present study. The difference may be attributed to the smaller male-to-female ratio of cheliped/shield length in *P*. *lanuginosus* compared to *P*. *filholi*, an inherent species-specific characteristic of the hermit crabs.

The parasitic effect size of rhizocephalans is likely to be determined by the energy burden that they impose on the host, since the parasite ensures its reproduction success by castrating or sterilizing the host to absorb the host’s reproductive energy [47]. Parasitic castrations often induce changes in the host’s behavior and metabolism [47, 48]. Energy extraction by rhizocephalans is thought to depend on number of eggs, egg size, number of breeding events per externa, and number of reproductive externae [19]. However, these factors have not been well-documented for *P*. *gracilis*, *P*. *postica*, and *P*. *lineata*. A different peltogastrid species *Peltogaster paguri*, which typically possesses one oval-shaped externa, undergoes 3–5 breeding events [49], with each event producing between several hundred and 28,000 eggs [49, 50]. Comparable numbers of eggs and breeding events per externae are anticipated for *P*. *postica* and *P*. *lineata* used in this study. In contrast, the breeding potential per externa remains unclear in peltogasterellids, which possess one or more externae. However, all externae possess reproductive potential, each capable of at least two breeding events [36]. Therefore, the amount of energy extracted by rhizocephalans from the host may vary between species, leading to differences in parasitic effect sizes on the host.

To investigate the relationship between the energy extracted through castration and molting suppression and the parasitic effect on the host, it is crucial to clarify the number of breeding events, number of eggs, egg size, and number of reproductive externae in peltogasterellids and Peltogastrids through long-term rearing experiments. Although Nagler et al. 2017 [19] suggest that host utilization varies among rhizocephalan species, few studies have compared parasitic effect sizes across multiple rhizocephalan and host species. The present study is the first to compare not only the effects of two rhizocephalan parasites, *P*. *gracilis* and *Peltogaster* sp., on host hermit crabs in two geographically distinct populations, but also the parasitic effect size between two host species, *P*. *lanuginosus* and *P*. *filholi*, parasitized by either *P*. *gracilis* or *Peltogaster* sp.

However, the lack of species-level identification of *Peltogaster* sp. in the present study limits the interpretation of these dynamics. Rhizocephalans have highly simplified external morphology, lacking distinct diagnostic characteristics, which has led to the reliance on histological surveys for species identification [39]. Additionally, unidentified *Peltogaster* sp., which cannot be distinguished based on external morphology and COI data, further complicates species identification in this study. To gain a more comprehensive understanding of the impacts of rhizocephalans on their host species, future studies should compare host–parasite relationships not only among Paguridae, *P*. *gracilis*, and *Peltogaster* sp., but also across other hermit crab hosts and rhizocephalan species.

Morphological feminization in hermit crabs caused by rhizocephalans has been documented for species such as *Peltogaster paguri* (family Peltogastridae) and *P*. *gracilis* (family Peltogasterellidae), which parasitize various Paguridae hosts, including *Pagurus pubescens*, *P*. *ochotensis*, and *P*. *pectinatus* [51]. A recent transcriptome study [52] suggests that rhizocephalan parasites may alter neurotransmitter secretion in the eyestalk and thoracic ganglia, leading to feminized morphology and behavior in male hosts. However, the molecular mechanisms underlying morphological feminization remain unclear. Future research should employ comparative transcriptomics of parasitized and unparasitized hosts, along with neurobiological analyses, including neuronal activity tracking and immunohistochemical labeling, to elucidate the mechanisms driving rhizocephalan–induced morphological feminization in hermit crabs.

## Conclusions

Rhizocephalans are significant parasites of decapods, including hermit crabs, crabs, and shrimps. This study is the first to compare the effects of the rhizocephalan parasites *P*. *gracilis* and *Peltogaster* sp. on host hermit crabs, *P*. *lanuginosus* and *P*. *filholi*, from two geographically distinct populations (Asari, Hokkaido, and Chikura, Chiba, Japan). The frequency of second pleopod appearance was consistently higher in males parasitized by *P*. *gracilis* than in those parasitized by *Peltogaster* sp. Furthermore, cheliped length was significantly reduced in males parasitized by *P*. *gracilis* compared to unparasitized individuals, whereas males parasitized by *Peltogaster* sp. exhibited no reduction in cheliped length in *P*. *lanuginosus*, although reductions were observed in *P*. *filholi*. This study demonstrates that *P*. *gracilis* and *Peltogaster* sp. differentially induce morphological feminization in their host. Additionally, parasitic effects were significantly different between *P*. *lanuginosus* and *P*. *filholi* parasitized by *P*. *gracilis*, whereas similar parasitic effects were observed between the two host species parasitized by *Peltogaster* sp. These findings indicate that the impact of rhizocephalan infection on host morphology varies by rhizocephalan and host species. Future research should compare host–parasite relationships across other hermit crab hosts and rhizocephalan species to better understand the mechanisms underlying these host–parasite interactions.

## Data Availability

The datasets during the current study are available from the corresponding author on request.
